# Development of ultra-low volume, multi-bio fluid, cortisol sensing platform

**DOI:** 10.1038/s41598-018-35199-5

**Published:** 2018-11-13

**Authors:** Sayali Upasham, Ambalika Tanak, Badrinath Jagannath, Shalini Prasad

**Affiliations:** Deparatment of Bioengineering, University of Texas at Dallas, Richardson, T.X-75080 USA

## Abstract

The development of a non-faradaic electrochemical sensor for screening across multiple bio-fluids that demonstrate the expression of cortisol using a gold microelectrode-based sensor is reported in this paper. Room temperature ionic liquid (RTIL), BMIM[BF_4_] was used as the buffer to modulate the electrical double layer (EDL) to enhance the electrochemical signal response of the sensor. The sensor design and the surface chemistry was optimized using COMSOL Multiphysics software simulations and FTIR respectively. The sensor was designed so that it uses ultra-low volumes between 3–5 µL of bio-fluid for detection. Cortisol detection was achieved in the physiologically relevant ranges when tested in serum, blood, sweat, and, saliva using non-faradaic Electrochemical Impedance Spectroscopy (EIS) and performance parameters of the sensor were determined. Sensor’s response was tested against the only commercially available salivary cortisol point-of-care kit using regression analysis. Cross-reactive studies using prednisone indicated that the sensor is specific for cortisol. The sensor displayed a correlation value i.e. R^2^ > 0.95 between the signal response and the concentration of cortisol present in the system. Dynamic range of the sensor was across the physiologically relevant range of cortisol i.e. 50–200 ng/ml for serum/blood, 1–40 ng/ml for saliva, and 10–150 ng/ml for sweat. Limit of detection for serum and sweat was 10 ng/ml and 1 ng/ml for saliva.

## Introduction

The past decade has witnessed a rise in adopting a patient centric approach for better overall healthcare management. This has led to a tremendous growth in the development of point-of-care (POC) devices to enable self-monitoring, thereby, providing an ease in patient care without being restricted to hospitals. In 2016, the global POC testing market was estimated at 16.7 billion USD and is projected to become 36.96 billion USD by 2021^1,2^. However, these devices have been limited to monitoring activity-related parameters such as tracking steps walked, calories burnt, heart rate tracking and blood pressure monitoring^3,4^. Most of the diagnostic devices still rely on conventional laboratory-based blood or serum testing that have several limitations such as prolonged processing times, large sample volume, higher cost, and patient discomfort. On the contrary, POC devices can be effective in disease management as they are minimally/non-invasive that require low sample volumes and perform rapid sensing of biomarkers. Furthermore, these POC devices are user friendly, convenient and facilitate self-monitoring which match the guidelines provided for improving patient-centric healthcare approach issued by the National Patient Safety Goals, The Joint Comission-2018^5^. Currently, the available POC diagnostic devices are limited to detecting biomarkers in a single body fluid, mostly blood.

Biomarkers such as cortisol are expressed in serum/blood, sweat and saliva making its detection in multiple bio-fluids of greater diagnostic relevance. Cortisol is a steroid hormone involved in regulating a wide variety of process throughout the body, primarily, regulating metabolism and immune response^6–8^. Additionally, cortisol is a well-known biomarker for stress^9^. It is known to follow a circadian rhythm, which alters cortisol levels throughout the day according to the sleep-wake cycle^10^. Increased levels of cortisol could lead to greater risk for diabetes, high blood pressure, compromised immune system and also development of Cushing’s disease^11^. Therefore, monitoring cortisol levels can be used to understand the body’s response to stress and promote better lifestyle. Although, several research groups have demonstrated cortisol detection in either blood, serum^12^, sweat^13^, saliva^14^ or urine^15^; limiting detection to a specific bio-fluid restricts the ability to collect detailed physiological information, thus, compromising on the quality of the diagnostic outcome.

Our group has previously published about development of a MoS_2_ nanosheet based cortisol biosensor, that also uses low volumes of analyte for sensing and has demonstrated good sensitivity for cortisol in sweat^16^. Also, Parlak *et al*. have demonstrated the development of a patch-type, Molecularly imprinted polymer (MIP) based wearable cortisol sensor that detects cortisol in the range of 0.1–1 µM^17^. Although, these papers demonstrate good sensitivity for cortisol, they have certain shortcomings. The latter work uses a volume of 50–100 µL of sweat and demonstrates sensitivity in a narrow range for cortisol. However, both of these papers do not demonstrate translatability across all bio-fluids. This is a challenge as the buffering capacity of different bio-fluids varies with its composition. In this work, we have demonstrated, a novel, ultra-low volume, universal bio sensing platform that can rapidly and reliably detect cortisol in serum, blood, sweat and saliva. In order to achieve a robust and highly sensitive sensing response, non-faradaic label-free electrochemical impedance spectroscopy (EIS) was used as the detection modality. However, the key challenges in developing a universal biosensor include retaining sensitive, stable and selective detection measurement for different bio-fluids being tested that differ in properties such as viscosity, ionic composition, pH and conductivity. Therefore, to improve the performance of the sensor in the presence of these varying bio-fluid matrix compositions, we incorporated the use of room temperature ionic liquids (RTIL).

RTILs have been previously known to be used to augment protein stability^18^. Owing to their favorable properties such as widespread electrochemical window, physio-chemical stability as well as high thermal stability, RTILs have found their use in many applications. Out of the many RTILs currently being researched, our group has previously demonstrated sensitive, stable and reliable protein detection for more than 24 hours by leveraging properties of 1-butyl-3-methylimidazolium tetrafluoroborate (BMIM[BF_4_]) in human sweat and serum^19,20^. BMIM[BF_4_] lies in the center of the Hofmeister series enabling protein stabilization. The cationic and anionic properties define the overall performance of the RTIL. The BMIM cation reduces the tendency of protein aggregation while BF_4_ anionic moiety prevents hydration of the protein^21^. Therefore, we can utilize the properties of BMIM[BF_4_] for improving bio-sensing of cortisol regardless of the buffer medium. Thus, the present work is the first demonstration of a novel, universal, label-free, electrochemical biosensing platform to detect cortisol across body fluids in a sensitive and reliable manner.

## Results and Discussion

Due to the varying characteristic physical properties (see Table 1) of human bio-fluids, the task of detecting biomolecules on a single sensor platform is challenging. Currently, no such universal platform exists that possess the capability to detect biomolecules across various human buffers. In this work, we leverage the electrochemical properties of BMIM[BF_4_] towards developing a universal sensor platform for bio-sensing cortisol across various bio-fluids such as serum, whole blood, saliva and sweat. Our research group has previously demonstrated the properties of BMIM[BF_4_] to enhance the stability and specificity of the sensor for detection of biomolecules in human sweat^19,20^. Hence, the developed electrochemical sensing platform is bio-fluid agnostic and demonstrates robust and reliable detection of cortisol across these bio-fluids.

**Table 1:** Properties of different bio-fluids and Phosphate buffered saline (PBS).

Parameter	Sweat	Saliva	Serum	Blood	PBS buffer
Conductivity (S/m)	0.2–1.1	0.4	1.18	0.5-0.6	1.5–2
Viscosity (cP)	0.8–1.0	1.68	1.5	3.2	1.05
pH range	3.5–8.0	4.0–8.0	7.0–7.4	7.35–7.45	7.4

This section is organized in the following manner (1) Optimal electrode design characterization using COMSOL Multiphysics simulation software; (2) Sensor electrochemical characterization and validation of immunoassay chemistry; (3) Electrochemical calibration of bio-sensing performance for detection of cortisol in human bio-fluids; (4) Evaluation of biosensor specificity and selectivity in the presence of cross-reactive biomolecules.

### Electrode design characterization using COMSOL Multiphysics software simulations

**Figure 1:**
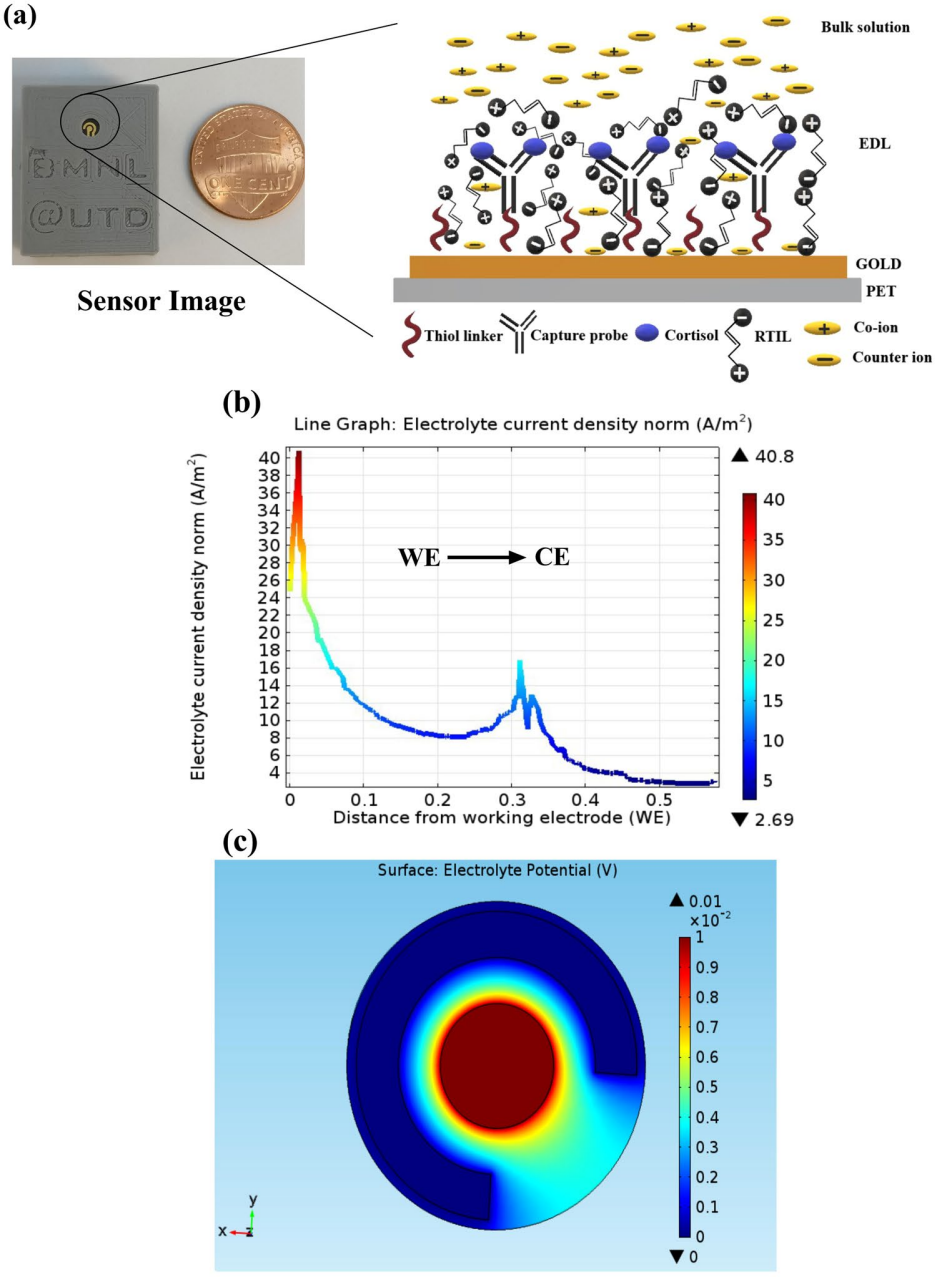
(**a**) Sensor image (left) indicating electrode sensing area scaled against one cent coin, schematic cartoon (right) depicting the electric double layer (EDL) electrochemistry in the presence of RTIL and after an external positive potential bias is applied to the electrode. (**b**) COMSOL Multiphysics software line plot for simulation of current density from working electrode (WE) to Counter electrode (CE). (**c**) COMSOL Multiphysics software plot for distribution of electrolyte potential.

A schematic of the electrode along with the geometry of the sensing region is depicted in Fig. 1a. Finite element analysis using COMSOL Multiphysics simulation software based on this sensor design was used to simulate the electrode behavior in the presence of an electrolyte and to optimize electrode design for enhanced performance^22,23^. These simulations were performed to gain insight of the electric field distribution of the biosensor in the presence of an electrolyte which dictate the bio-sensing performance of the system. Figure 1a (see inset) depicts the phenomenon of double layer formation of the electrode in the presence of an electrolyte (RTIL) and a positive potential bias being applied to the system. EDL in the figure represents the ‘electrical double layer’ which has a capacitive behavior and is modulated whenever there is binding between the antibody i.e. capture probe and the cortisol molecules on the functionalized gold microelectrode surface. The COMSOL Multiphysics model was developed for this gold microelectrode on a polyethylene terephthalate surface (PET) with an electrolyte layer of BMIM[BF_4_] on the electrode. Surface current density and electrolyte potential were simulated using a physics-controlled mesh to determine surface-electrolyte potential difference and charge distribution at the electrode- electrolyte interface. The electrode boundary conditions were applied to the circular cross-section of the electrode and counter/reference electrode was ground/insulated. A potential of 10 mV was applied to the working electrode and Neumann’s boundary condition (n. J = 0) was applied to the electrolyte layer (see Supplementary Fig. S1a). The equations that govern the simulated potential and current density are listed in the supplementary.

The results of the simulation demonstrate current density and electrical field distributions as shown in Fig. 1b,c respectively. The output current from WE vary from 40 A/m^2^ to 0 A/m^2^ in the direction from the working electrode towards the counter electrode. From the line plot of the current density shown in Fig. 1b, maximum current density is observed around the working electrode which is 40 A/m^2^ and drops to zero at the counter electrode. A charge density of 20 A/m^2^ is observed around the counter electrode region, it still less than the maximum current density at the working region. According to the color-coded gradient scale in the figure, the red region corresponds to maximum current density which is observed only around the working electrode. The electric field is confined within the electrode and the electrolyte medium and maximum electrolyte potential is obtained around the working electrode (i.e. WE) (Central circle) which is chosen for immunoassay functionalization. The potential gradient is 10 mV at the working electrode and drops to zero mV at the counter electrode as shown in Fig. 1c. We infer from these simulations that the higher electric field distribution and current density around the working electrode makes it suitable for generating enhanced electrochemical response. The higher electric field and current density distributions are driven mainly by the gold microelectrode and RTIL interface making it a suitable design for developing a universal bio-sensing platform. The simulations were carried out on a scaled down model also (See Supplementary Fig. S1b,c), that indicated that the potential and current density remain constant. Hence, for practical issues, this size and design was determined to be the best fit for building this cortisol sensing platform.

### Sensor electrochemical characterization and validation of immunoassay chemistry

#### Surface wettability characterization

One of the desirable characteristics of the biosensor should be its surface compatibility with the substrate which is determined through its wetting properties. The bio-fluid of interest on interaction with the substrate should uniformly wick across the electrode surface^24^ (see Supplementary Fig. S1d). Contact angle studies were performed to validate wettability (see Supplementary Fig. S1e). A contact angle of 62–64° indicates the hydrophilic nature of the substrate. Low-sample volumes in the regime 1–10 μL are advantageous for point-of-need diagnostics. In this study, we utilize 5 μL sample volume for bio-sensing. To ensure uniform wicking of low volume of sample fluid, surface modification technique such as plasma treatment was adopted to further enhance the hydrophilicity of the substrate. The hydrophilicity of the substrate is confirmed by the drop in contact angle to 29–30^24^.

#### Open circuit potential measurements for determining biosensor stability

**Figure 2:**
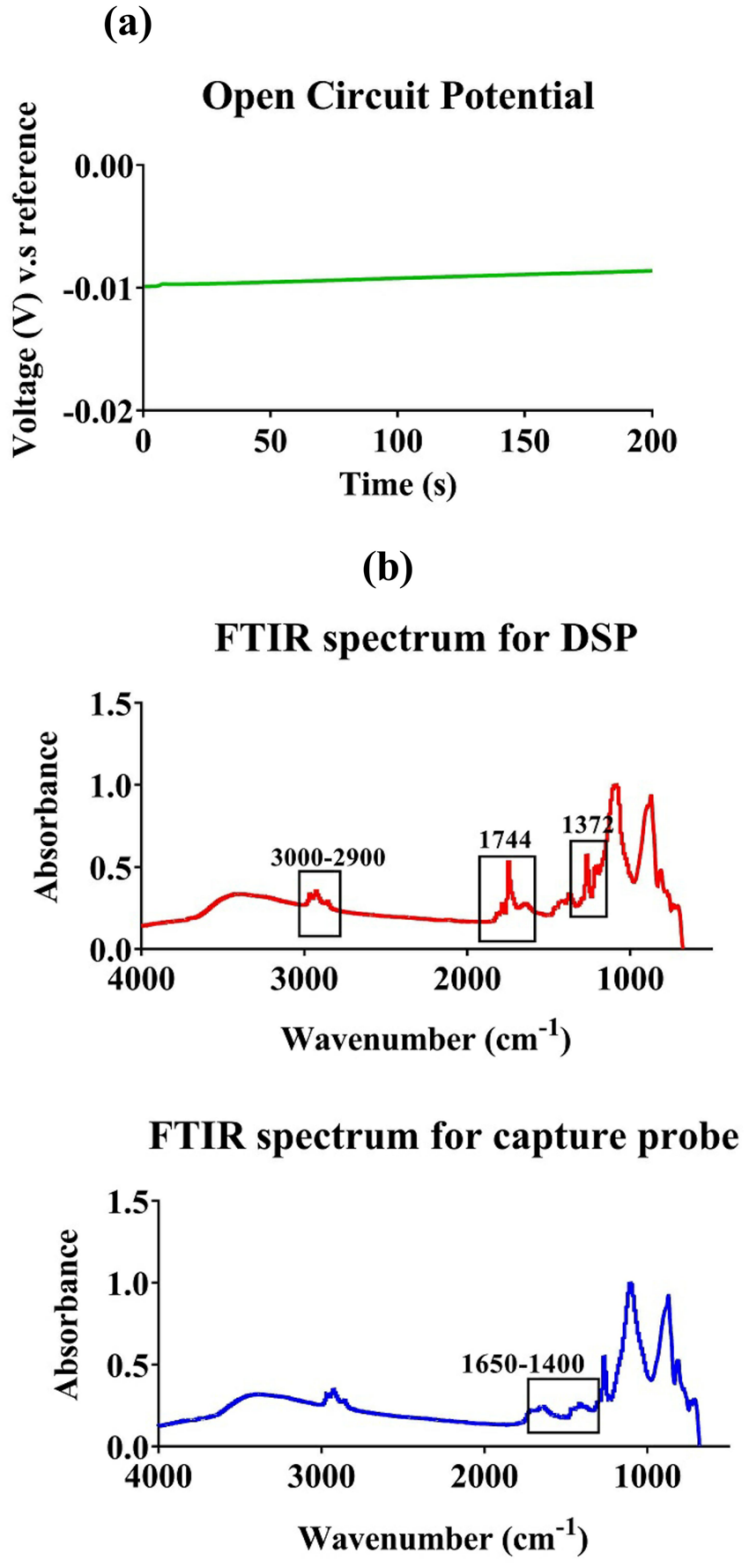
(**a**) Open circuit potential measurement for the sensor is stable for over 200 s. (**b**) FTIR spectra for immobilized thiol linker DSP (top) and capture probe-cortisol antibody (bottom) on the electrode surface.

The biosensor design required for robust bio-sensing was determined from the electrical field and current density plots obtained from the COMSOL simulations. Further baseline electrochemical characterization was performed to evaluate electrochemical stability of the biosensor platform for operation in the human bio-fluid panel under study. Open- circuit potential (OCP) determines the inherent potential gradient associated between electrodes in the presence of an ionically charged buffer^25^. Typically, a stable electrochemical sensor should have an OCP in the lower millivolt range when measured over time. As represented in Fig. 2a, an OCP of 10 mV was attained over 200 s indicating the electrochemical stability of the sensor.

#### FTIR analysis

Fourier transform infra-red spectroscopy (FTIR) was utilized to validate functionalization of the immunoassay chemistry on the biosensor electrode surface and to ensure appropriate cross-linking of the immunoassay components. FTIR spectroscopy was performed to confirm the successful immobilization of the cortisol antibody (capture probe) with the DSP cross-linker (Dithiobis [Succinimidyl Propionate]) functionalized on the gold electrode surface. First, the functionalization of DSP cross-linker on the electrode surface was characterized. IR spectra for the linker and the antibody are depicted in Fig. 2b. Table 2 lists the relevant peaks obtained after immobilization of DSP and cortisol antibody.

**Table 2:** FTIR peak positions indicating the presence of Self-assembled monolayer for the biosensor.

Description	Expected Peak position	Peak position obtained
Stretching of CH alkane chain	3409 cm^−1^	3000 cm^−1^
Symmetric carbonyl stretch of NHS in DSP linker	1745 cm^−1^	1744 cm^−1^
CH_2_ bending	1431 cm^−1^ and 1456 cm^−1^	1458 cm^−1^
CH_3_ bending	1372 cm^−1^	1372 cm^−1^
Amide-I bond	1500–1550 cm^−1^	1558,1540,1506 cm^−1^
Amide-II bond	1652 cm^−1^	1652 cm^−1^

The characteristic peak indicating the presence of the DSP linker is at 1744 cm^−1^. This peak indicates the symmetrical carbonyl stretch (C=O) of the NHS ester. DSP has an alkane chain present that is visible as sharp peaks at 2923 cm^−1^ and 2962 cm^−1^. These respective peaks correspond to asymmetric and symmetric stretching occurring in the methylene groups of the alkyl chain^26^. Following antibody functionalization on the DSP immobilized surface, we can observe a decrease in the 1744 cm^−1^ peak which indicates breakage of the CO-NHS bond due to binding of antibody to the DSP linker. Furthermore, the binding of the antibody results in peaks corresponding to the two amide bonds-Amide- I and Amide- II of the antibody as described in Table 2. Details about the bonds corresponding to these amide peaks and other antibody specific peaks has been described in the supplementary. Thus, the FTIR results confirm the successful immobilization of cortisol antibody on the sensor surface.

### Electrochemical calibration of bio-sensing performance for detection of cortisol in human bio-fluids

After confirming successful immobilization of the capture probe on the electrode surface, the sensor’s performance for detection of cortisol in bio-fluids (serum, whole blood, saliva and sweat) was evaluated using non-faradaic EIS. This method can be used to detect the target biomolecule directly without the need to use a redox label, which is typically required in other electrochemical techniques. Furthermore, non-faradaic EIS captures subtle changes due to binding interactions within EDL at the electrode/solution interface, thus resulting in a highly sensitive response. These binding interactions are typically represented as EDL capacitance modulation. As discussed earlier, the sensor EDL schematic is demonstrated in Fig. 1a. On application of an AC voltage perturbation, capacitance modulations in this EDL layer occur whenever there is binding between the cortisol molecule and the anti-cortisol antibody. These modulations are directly captured using EIS and analyzed as Nyquist and Bode plots^25^. Also, this EDL layer can be simulated electrically using the Randle’s circuit, In this work, we have modeled the data using a modified Randle’s circuit which has been described in the supplementary. Furthermore, we have leveraged the properties of RTIL, BMIM[BF_4_], to obtain an enhanced sensitive response for detection of cortisol across different bio-fluids. The performance in invasively obtained bio-fluids like serum and whole blood is characterized followed by non-invasively obtained fluids such as saliva and sweat.

#### Analysis of performance in serum and whole blood

**Figure 3:**
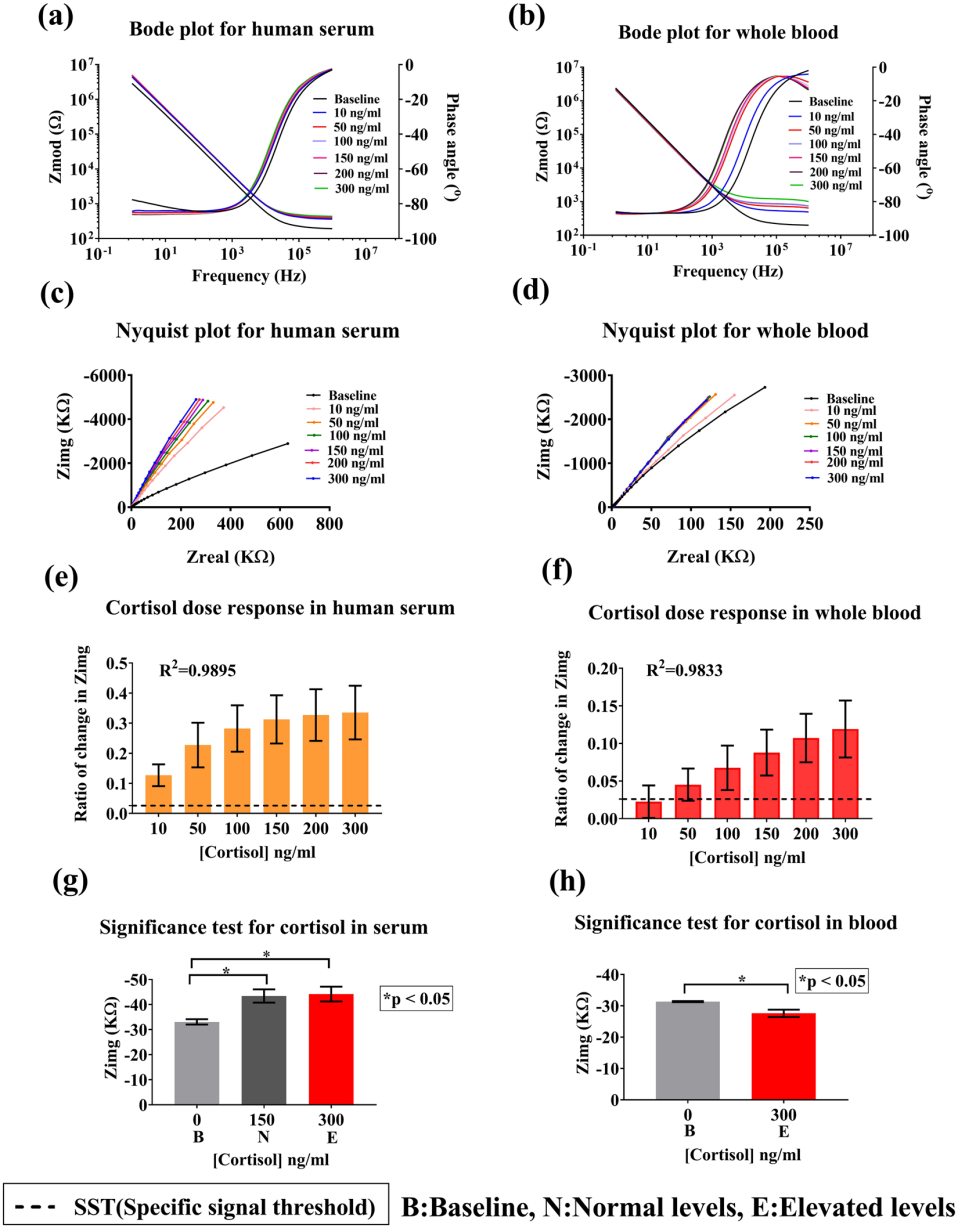
Analysis of sensor performance in serum and blood. (**a**) Bode phase and magnitude plot for human serum. (**b**) Bode phase and magnitude plot for whole blood. (**c**) Nyquist plot for human serum. (**d**) Nyquist plot for whole blood. (**e**) Calibration dose response of cortisol spiked in serum plotted as change in imaginary impedance (Zimg) from baseline. (**f)** Calibration dose response study for cortisol spiked in whole blood, **(g**,**h**) Statistical analysis for significance in impedance change from baseline for (**g**) serum and (**h**) whole blood.

The physiologically relevant range for cortisol in serum and whole blood is 20–250 ng/ml^7^. In addition to cortisol being present, whole blood has a complex mixture of erythrocytes, leukocytes, thrombocytes and plasma, and a high amount of proteins and steroids or hormones. Direct bio-sensing is challenging in this case because of this highly complex non-Newtonian fluid behavior of blood^27^. Therefore, the use of BMIM[BF_4_] helps in enhancing the signal response by selectively allowing cortisol to bind to the immobilized capture probe. The detection modality used here is EIS and the dose dependent response of the sensor is recorded as impedance changes. Impedance data is analyzed and represented as Bode and Nyquist plots in Fig. 3a–d. These plots are the frequency signatures of the bio-sensing system in response to cortisol-cortisol antibody binding. The Bode phase plot represented in Fig. 3a,b for cortisol spiked in serum and whole blood shows a maximum capacitive phase of 85–90^o^ at the lower frequency range (i.e. between 1–1000 Hz) due to the capacitive binding of the target cortisol molecule. In the higher frequency regime (10000 Hz-1 MHz) a resistive response is observed (phase angle <45^o^) which is due to the bulk behavior of the electrolyte system^28,29^. The Nyquist plot for serum and whole blood is represented in Fig. 3c,d. For serum and blood, with the increase in the concentration of cortisol, the radius of curvature of the plot decreases and is closer to Zimg of the impedance. The binding of cortisol molecules to the antibody results in an increased change of the EDL capacitance, thus causing a change in Zimg. Typically, the binding interactions within the EDL are captured at the lower frequencies (<1000 Hz) in these impedance plots^15,25^.

Comparison of electrochemical response for cortisol detection in presence of RTIL and an aqueous buffer was carried out to evaluate the signal enhancement contributed by RTILs (see Supplementary Fig. S3). The maximum impedance change using the cortisol antibody in aqueous buffer was 4.6% ± 2% which was in contrast to the maximum impedance change of 33% ± 8% with the RTIL. This change is calculated from the baseline value i.e. zero dose (blank dose without cortisol). The mechanism through which RTILs enhance the signal response by the formation of a multilayer EDL at the electrode-RTIL interface when the electrode is polarized. When there is an increase in the charge density at the surface of the electrode due to binding events occurring between the cortisol molecule and the capture probe, the cation-anions stretched out to form multiple stacks of cation- anion pairs. Due to the electrostatic interactions occurring between the cation, anion of the RTIL and the charged electrode surface, the EDL instead of being a single layer consists of a multilayer in order to compensate for the increased charge density at the electrode surface^30,31^. This multilayer EDL thickness is determined to be 10–12 ionic layers^32^. The formation of multilayer EDL by the RTIL prevents charge screening, thereby enhancing signal response of the sensor, when compared to the response in aqueous buffers. Thus, making it a suitable buffer choice for signal enhancement of this sensor^33^.

Selection of an optimal working frequency is important to determine the performance parameters of the sensor. The maximum change in impedance from baseline was obtained at 100 Hz. Also, from bode phase plot, it was determined that the maximum capacitive phase response occurs at 100 Hz corresponding to the binding interactions of cortisol. Thus, this frequency was chosen for evaluating the sensor performance. At this frequency, due to the capacitive nature of the sensor, the imaginary component of the impedance (Zimg) has a dominating influence on the impedance behavior. Thus, the calibration dose responses have been calculated using Zimg values. Figure 3e,f depict the calibrated dose response (CDR) of the sensor for various concentrations of cortisol spiked in serum and blood. The Y axis represents the ratio of change in Zimg from baseline (which is the blank zero dose) and the X axis signifies cortisol concentration in the range of 10–300 ng/ml. The ratio of change in Zimg varies from 12.6% ± 3% to 33.5% ± 8% for increasing concentrations of cortisol in serum and from 2.2% ± 2% to 12% ± 3% for whole blood. This change is due to an increased charge carrier density with the increase in cortisol biomolecules binding to the antibody functionalized the electrode surface. The black dotted line is the specific signal threshold (SST) as described earlier. A limit of detection (LOD), which is the minimal concentration that can be detected above SST, of 10 ng/ml was obtained in serum and 50–100 ng/ml in blood. Fig. S2a,b represent the regression curve-fit analysis (see supplementary). A R^2^ of 0.98 was obtained for both saliva and serum. Furthermore, an unpaired two-tailed t-test was performed to test for statistical significance of the sensor to distinguish the signal response to cortisol from baseline and, distinguish normal and elevated levels of cortisol. Statistical significance of p < 0.05 was obtained with a confidence interval (C.I.) of 95% as indicated in Fig. 3g,h. Thus, the sensor can reliably distinguish between normal and elevated cortisol levels.

#### Analysis of performance in sweat and saliva

Cortisol levels in bio-fluids like sweat and saliva have good correlation with well characterized bio-fluids like serum and blood^34^. In addition to being convenient sources of self-monitoring, these bio-fluids contain free cortisol which can cross membrane barriers easily. These bio-fluids give a measure of this biologically active cortisol present in the human body that correspond to illness severity. Also, as these bio-fluids are easily accessible, continuous monitoring of the circadian patterns of cortisol becomes easier. Thus, estimation of cortisol from non-invasive bio fluids serve greater importance in diagnostics^35^.

Human saliva: Salivary cortisol provides us an estimate of the pharmacologically active form of cortisol^7^. Also, salivary cortisol levels have a good correlation with serum cortisol levels as described by Dorn *et al.*^34^. It aligns with the circadian rhythm of cortisol in the body and has cortisol level amplitudes coinciding with those of serum indicating that the cortisol levels in saliva directly reflect the serum levels across a 24 hour period. Thus, monitoring of circadian rhythm of cortisol in saliva is of high diagnostic value.

**Figure 4:**
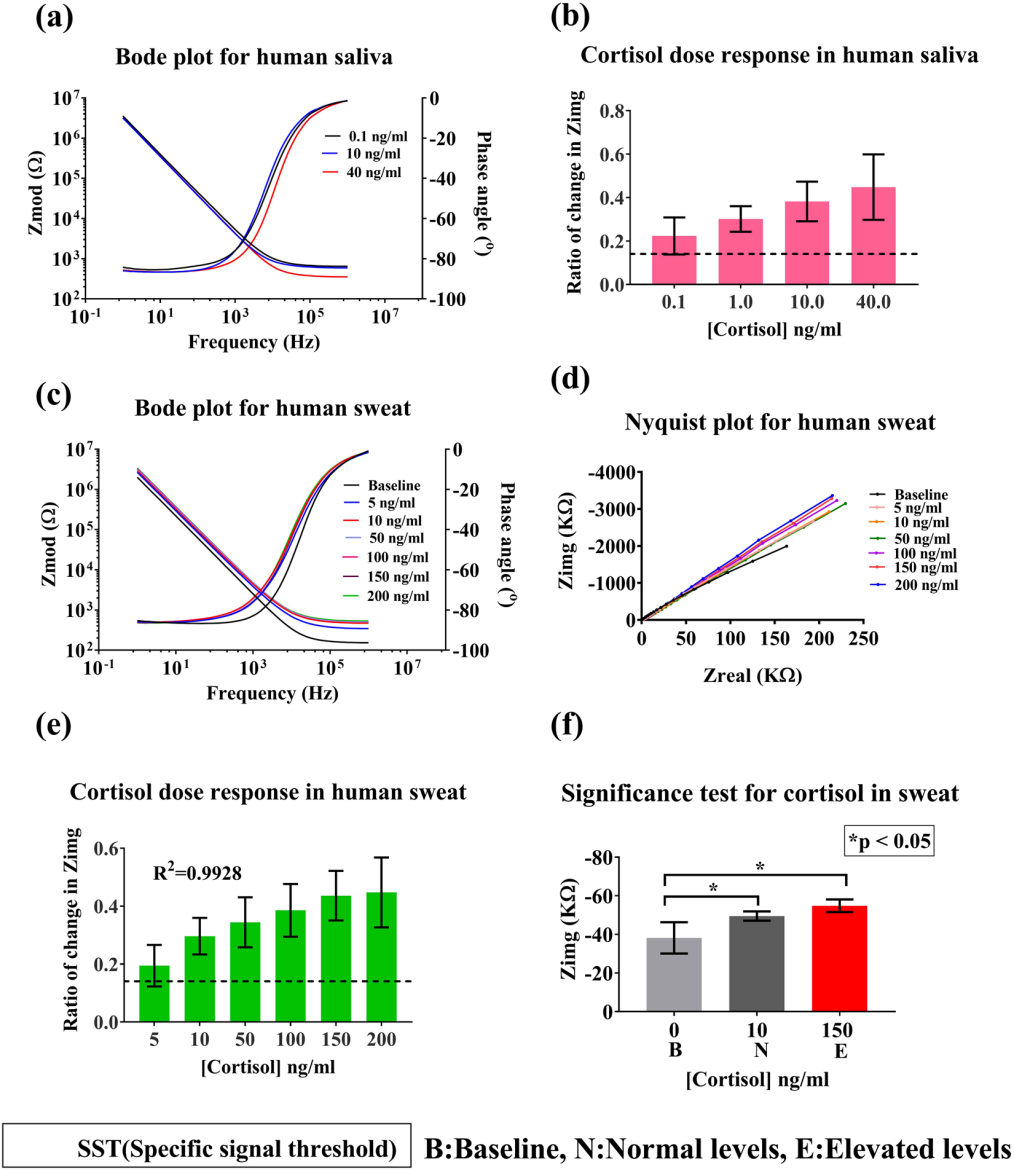
Analysis of sensor performance in saliva and sweat. (**a**) Bode phase and magnitude plot for human saliva. (**b**) Calibration dose response of cortisol spiked in saliva plotted as change in imaginary impedance (Zimg) from baseline against cortisol concentration. (**c**) Bode phase and magnitude plot for human sweat. (**d**) Nyquist plot for human sweat. **(e)** Calibration dose response study for cortisol spiked in human sweat. (**f**) Statistical analysis for significance in impedance change from baseline for sweat.

The physiologically relevant range of cortisol in human saliva is 1–30 ng/ml with normal ranges between 1–10 ng/ml^12^. The sensor was tested for its ability to detect cortisol levels in the range 0.1–40 ng/ml. This is the first report of the use of RTIL based cortisol detection platform for obtaining an enhanced signal response for cortisol in saliva. The ionicity and pH of saliva may largely impact the electrochemical signal response. Therefore, multiple blank saliva (without cortisol) washes was performed to evaluate if the ionic nature of saliva contributed to any impedance change. The control study showed no change in impedance even after multiple washes (see Supplementary Fig. S4a). The stability of the sensor i.e. not being susceptible to any ionic changes, is primarily maintained by the RTIL, which prevents any ionic interactions at the electrode/solution interface. Hence, the developed sensor is suitable for biosensing in buffers with varying pH and ionicity. Figure 4a represents the bode magnitude and phase plot, showing the change in impedance response with varying concentrations of cortisol in saliva. A maximum capacitive phase response was observed at 100 Hz, similar to the serum and whole blood results. CDR curve for saliva is depicted in Fig. 4b. The maximum change in impedance from baseline for varying cortisol concentrations is 44% ± 15% for 40 ng/ml cortisol. A LOD of 1 ng/ml was obtained with an SST of two times the standard deviation of baseline blank buffer. This indicates that the sensor can reliably detect low concentrations of cortisol in saliva. The high sensitivity can be attributed to the role of BMIM[BF_4_] that allows for the selective binding of cortisol, forming a shield around the cortisol antibody, thus, preventing non-specific molecules/ions from modifying the electrode/solution interface^2,20^.

Human sweat: Human eccrine sweat is an attractive bio-fluid for self-monitoring cortisol levels using wearable technology. Thus, The sensor was tested for detection of cortisol concentrations (5 ng/ml to 200 ng/ml) in human sweat which has a physiological relevant range between 8 ng/ml and 141 ng/ml^19^. Bode phase and magnitude plots illustrated in Fig. 4c show a dose dependent distinctive capacitive behavior of the sensor. As the cortisol concentration increases from 5 ng/ml to 150 ng/ml, there is a decrease in the Zimg component of the impedance observed which is reflective of increase in the overall capacitance of the double layer at each immunoassay step. Phase shift of −90 degrees can be observed from bode phase plot for frequencies corresponding to the double layer. From the Nyquist plot depicted in Fig. 4d, we can observe that the plot is an incomplete semicircle representing a typical non-faradaic response. Calibration dose response is represented in Fig. 4e. Zimg changed from 19% ± 7% to 45% ± 12% from baseline with increasing cortisol concentration. Limit of detection for the sensor is 10 ng/ml and the dynamic range is between 10 ng/ml to 200 ng/ml. A R^2^_=_ 0.99 was obtained as represented in Fig. S2c (see supplementary). Similar to the serum/blood signal response, significance was computed using an unpaired t-test described in Fig. 4f. This resulted in p < 0.05, for normal and elevated levels of cortisol.

**Figure 5:**
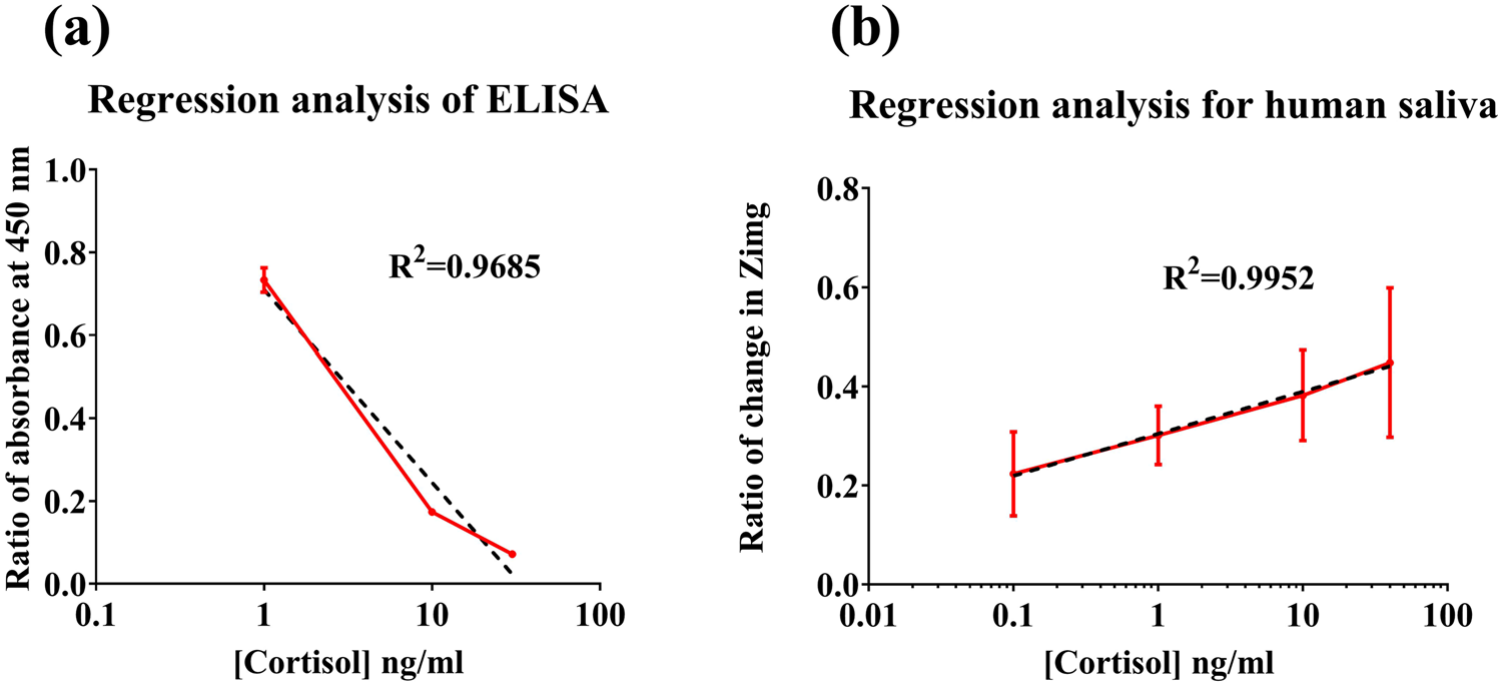
Comparison of sensor performance with commercially available technique -ELISA. (**a**) Regression analysis of sensor performance in saliva and (**b**) Regression analysis of data obtained from ELISA kit.

ELISA correlation for salivary cortisol: A correlation study was carried out using a standard commercially available salivary cortisol ELISA (Salimetrics, State college, PA, USA). This is depicted in Fig. 5, ELISA was performed using human saliva samples and three points were picked in the physiologically relevant range, to signify high, low and medium cortisol levels. Ratio of absorbance measured at 450 nm was calculated by generating a human saliva calibration curve for cortisol. There is a dose dependent trend for cortisol observed. Nonlinear regression analysis was carried out on the sample in order to fit a model. It resulted in a R^2^ of 0.97 depicted in the Fig. 5a. This data was compared with the sensor’s dose response data illustrated in Fig. 5b which has an R^2^ = 0.99. This indicates a linear dose dependency of change in impedance to the cortisol concentration and a better correlation for the two variables- cortisol concentration and the ratio of change in impedance. Thus, the performance of the sensor in human saliva was validated by comparing it with the commercially available techniques.

### Evaluation of biosensor specificity and selectivity in the presence of cross-reactive biomolecules

**Figure 6:**
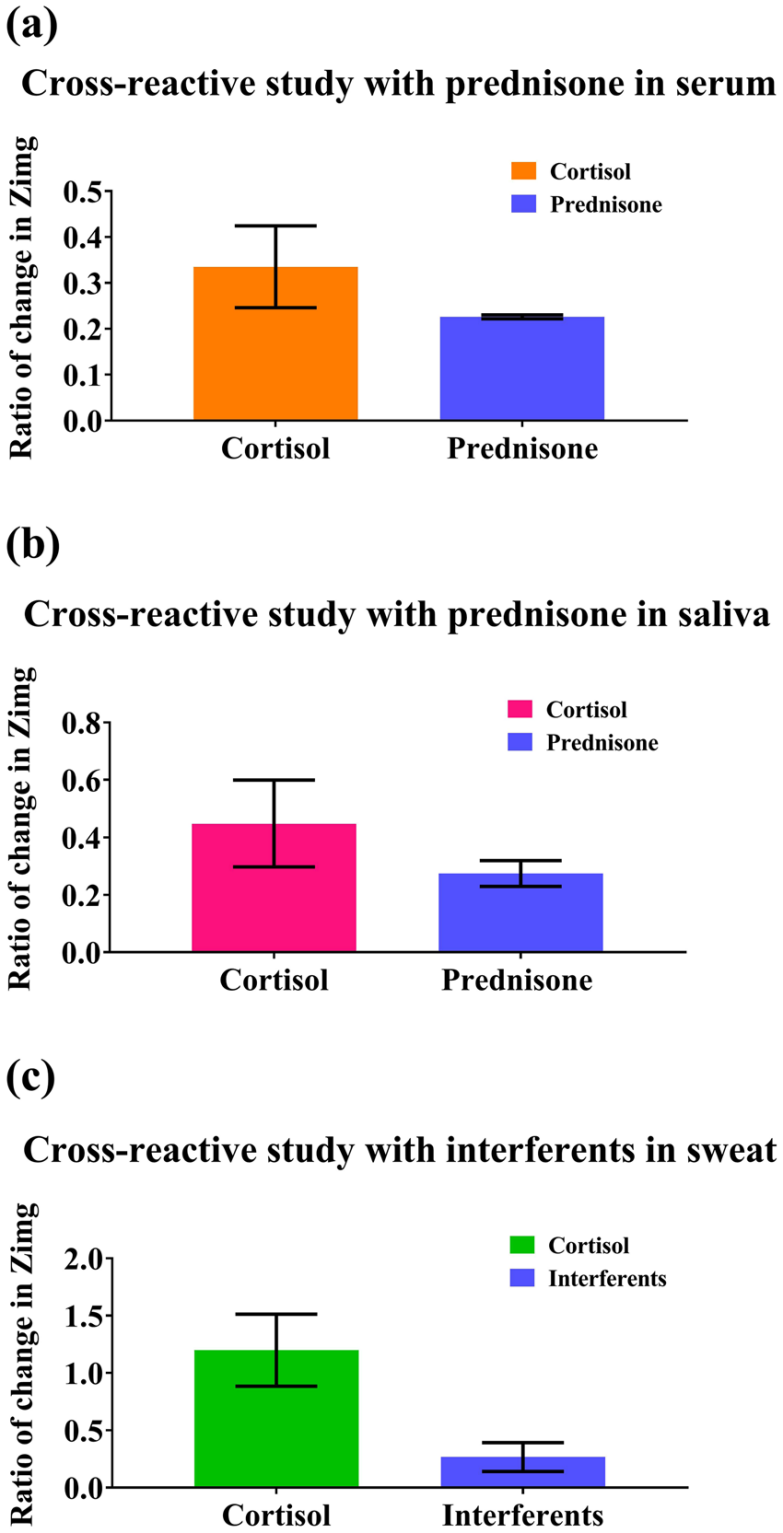
Cross-reactive studies in serum, saliva and sweat. (**a**) Cross-reactive study for sensor response in serum compared against sensor response for prednisone. (**b**) Cross reactive study for sensor response in saliva compared against sensor response for prednisone. (**c**) Cross-reactive study in sweat compared against sensor response for interferants.

The sensor was tested for its specificity for cortisol molecule and to understand the effect of cross-reactive agents on the signal response. Non-specific binding interactions affect the sensor response leading to generation of false positives. Prednisone has a similar structure to cortisol, and therefore, was used as a cross-reactive agent for testing the ability of the sensor to reliably detect cortisol. The sensor was tested against highest physiologically relevant concentration of prednisone in order to check for cross-reactivity. From Fig. 6a,b, it is evident that the maximum change in impedance for cortisol in serum and saliva is higher than that of prednisone. However, sweat contains other interferent molecules at high concentrations which may interfere with the electrochemical signal response. To quantify the signal generated by the interferents alone, a synthetic sweat interferent solution (pH 6) which simulates the behavior of human sweat was created. This interferent solution^36^ is a combination of small molecules and metabolites like glucose, lactate, creatinine, ascorbic acid and uric acid, which are present in human sweat. This study is represented in the Fig. 6c. The impedance change of the interferents alone was lower when compared with the impedance response for cortisol along with interferents, which indicates that the sensor could reliably distinguish cortisol from the interferent molecules. Although, there is some response observed for the cross-reactive agents, they contribute to the variability observed in the sensor’s response. Even in the presence of this variability, the sensor depicts a linear response for cortisol with the ability to distinguish between different levels of cortisol and other cross-reactive agents. Non-specific cortisol binding to the surface was also tested which resulted in response for non-specific doses to be below the SST line (See Supplementary Fig. S5). Thus, indicating that the sensor response is driven by binding events between the capture probe and the cortisol molecule. From these cross-reactivity studies, it can be confirmed that the sensor is specific for cortisol molecule and that the presence of interferent molecules does not affect the electrochemical detection ability of the sensor.

## Summary

We have leveraged the electrochemical properties of BMIM [BF_4_], towards the amplification of electrochemical signal response, in order to develop a robust, sensitive, flexible gold microelectrode-based biosensor for detecting cortisol across different bio-fluids. The signal response for non-invasively obtained bio-fluids and the invasive bio-fluids is comparable even though these bio-fluids have different physical properties which indicates that a single sensor platform can be used to perform robust electrochemical detection across bio-fluids with ultra-low sample volumes. This makes it desirable as a point-of-need system for monitoring cortisol levels. In this work, we have successfully demonstrated that the developed immunosensor is able to detect cortisol in the physiologically relevant range in human serum, whole blood, sweat and saliva with R^2^ values above 0.95. The biosensor exhibited the ability to distinguish between the different cortisol concentrations with respect to change in impedance. A comparison of this sensor with other cortisol detection technologies has been tabulated in Table 3. The primary factor for achieving a highly sensitive response can be attributed to the interaction behavior of BMIM[BF_4_] on the sensor surface. Due to charge interaction between the protein and BMIM[BF_4_], and their good charge retention properties, RTILs help in widening the electrochemical window at which the sensor operates, thus, making it possible for the electrode to have an enhanced signal response and ability to detect lower concentrations of biomarkers in fluids such as human saliva and sweat. The control studies performed confirmed the advantage of using an ionic-aqueous environment vis-à-vis an aqueous environment for electrochemical detection. The electrochemical detection ability of the biosensor was validated in the presence of cross-reactive molecules and interferents. The sensor performs robustly in the presence of interferents that could have produced a non-specific binding response. The use of this flexible hybrid biosensor makes a path for the development of an RTIL based, universal, non-faradaic electrochemical biosensor that can detect cortisol across different bio-fluids.

**Table 3:** Comparison of this work with other sensors.

Type of sensor	Limit of detection	Dynamic range	Volume	Bio-fluid	References
Surface plasma resonance (SPR) based sensor	0.36 ng/ml	1.5–10 ng/ml	25–50 μl	Saliva	^37^
Protein G based electrochemical immunosensor	16 pg/ml	50–2500 pg/ml	10 μl	Serum/Blood	^38^
Flexible nanoporous polyamide	1 ng/ml	10–200 ng/ml	3–5 μl	Sweat	^39^
Chemiluminescent-lateral flow immunoassay-based sensor	0.3 ng/ml	0.3–60 ng/ml	25 μl	Saliva	^40^
Flexible EIS based sensor	10 ng/ml-serum 1 ng/ml-saliva 10 ng/ml-sweat	Physiologically relevant range for each bio-fluid	5 μl	Serum Saliva Sweat	This work

## Methods

### Sensor fabrication

The flexible gold microelectrode sensors were fabricated using the technique of Cryo E-beam evaporation of a gold target on a flexible PET (Polyethylene terephthalate) surface in the clean room facility at The University of Texas at Dallas. The geometry of circular co-centric counter (external radius: 1.16 mm) and working electrode (radius: 0.38 mm) is designed in such a way that there is a proper size ratio maintained between the two electrodes. This reduces the amount of parasitic current generated which could affect the signal response of the sensor. Overall length of the sensor is 15 mm which makes it suitable for low analyte volume detection such as 3–5 μl, hence it is referred to as “microelectrode”. The design along with the dimensions is depicted in Fig. S6. Capture probes are immobilized on the working electrode and the immunosensing is carried out after applying a potential bias to the working electrode of the biosensor. Acrylic sheet-based shadow masks with this design imprinted were used for depositing gold. Rate of deposition was around 0.9–1.0 Å/s to achieve a final thickness of 250 nm of gold on the substrate. The inert nature of gold and the ability to use the linker electrochemistry in order to perform detection made it an ideal electrode material choice for this study. The potential setup of the sensor is depicted in Fig. 1a. The sensor is designed in such a way that it requires ultra-low volumes of analyte for detection.

### Reagents and materials

DSP (Dithiobis [Succinimidyl Propionate]), which is the linker used in this research, its solvent Dimethyl Sulfoxide (DMSO) and PBS (Phosphate buffered saline) were ordered from Thermofisher scientific Inc. (Waltham, MA, USA). Human serum was also purchased from Thermofisher scientific. Cortisol (Hydrocortisone) and the monoclonal α-cortisol antibody were ordered from Abcam (Cambridge, MA, USA). Whole blood was collected from Carter blood care (Plano, TX, USA). Human sweat was obtained from Lee bisolutions Inc. (St. Louis, MO, USA). BMIM[BF4] was purchased from Sigma-Aldrich (St. Louis, Mo, USA). Milipore DI water (Conductivity-18 MΩ.cm) was used for making the dilutions and the buffers. The antibody was diluted in BMIM[BF_4_].

### COMSOL Multiphysics software simulations

Finite element analysis was carried out using licensed version of COMSOL Multiphysics software 5.0 and using the primary current distribution physics (siec) under the Electrochemistry module. 3D plot group for electrolyte potential and 1D current density plot was used to describe the field and current density distribution for the simulated electrode model. Boundary conditions and governing equations are described in the supplementary.

### Experimental setup for FTIR

The Fourier transform infrared spectrum for capturing the binding between thiol linker and capture probe was recorded using Thermo scientific Nicole iS50 FTIR in Attenuated Total Reflectance (ATR) mode. The tool is equipped with a germanium ATR crystal, Deuterated triglycine sulfate (DTGS) detector and a KBr window. The sample was prepared on a glass substrate with washing and drying steps in between immobilizations to remove physiosorbed molecules. The spectrum was recorded between 675 cm^−1^ and 4000 cm^−1^ with a resolution of 0.5 cm^−1^ and 256 scans.

### Electrochemical Impedance spectroscopy

Whole blood, Human serum, sweat and saliva were spiked with cortisol and tested using Electrochemical Impedance spectroscopy (EIS). This calibration dose response study was performed in the frequency range of 1 Hz to 1 MHz with a potential AC bias of 10 mV applied to the electrode. Measurements were recorded using a Gamry Reference 600 potentiostat (Gamry Instruments, PA, USA). Calibration was performed using n = 3 samples. 10 mM of DSP linker was immobilized on the surface of the sensor for incubating for 1.5 hours. Following this 10 μg/mL of anti-cortisol antibody was immobilized on the electrode for 1.5 hours which was determined after a saturation study described in supplementary. After successful immobilization of the immunoassay on the sensor surface, the bio-fluids were dispensed on the surface and the impedance was measured using EIS. This was the baseline measurement as discussed in the results section. Post these measurements, various bio-fluids were spiked with cortisol in their respective physiologically relevant ranges in order to generate the calibrated dose response curves. All the samples were handled in accordance with the IRB protocol approved by The University of Texas at Dallas.

### Statistics

Data is represented as means ± SEM. Statistical significance was tested by performing unpaired two tailed t-test. This was performed on the calibrated dose response data for human serum, sweat and whole blood to indicate the ability of the sensor to distinguish normal and elevated levels of cortisol from the baseline, which is the blank dose. Non-linear regression and statistical analysis were performed using the statistical software Graph Pad Prism version 7.03 (Graph Pad Software Inc., La Jolla, CA, USA).

## Electronic supplementary material

Supplementary Information

## References

[1] *Point of Care (POC) Diagnostics/Testing Market Size, Share & Trends Analysis Report By Product (Glucose Testing, Hb1Ac, Coagulation, Fertility, Cardiac Markers, Hematology, Urinalysis) End-use, And Segment Forecasts* (2018).

[2] Marketsandmarkets.com. *Point-of-Care/Rapid Diagnostics Market by Testing (Glucose, Lipids, HbA1c, HCV, HIV, Influenza, Urinalysis, Hematology, Cancer, Pregnancy, PT/INR), Platform (Lateral Flow, Immunoassay), Mode (Prescription, OTC), End-User - Global Forecast to 2022* (2018).

[3] Cadmus-Bertram L, Marcus BH, Patterson RE, Parker BA, Morey BL (2015). Use of the Fitbit to Measure Adherence to a Physical Activity Intervention Among Overweight or Obese, Postmenopausal Women: Self-Monitoring Trajectory During 16 Weeks. JMIR mHealth uHealth.

[4] Ginsberg BH (2013). Practical Use of Self-Monitoring of Blood GlucoseData. J. Diabetes Sci. Technol..

[5] Joint Commission on Accreditation of healthcare organizations. Joint Commission announces national patient safety goals. Available at, https://www.jointcommission.org/standards_information/npsgs.aspx (Accessed: 23rd May 2018) (2018). 17682787

[6] de Kloet ER, Joëls M, Holsboer F (2005). Stress and the brain: from adaptation to disease. Nat. Rev. Neurosci..

[7] Gatti R (2009). Cortisol assays and diagnostic laboratory procedures in human biological fluids. Clin. Biochem..

[8] Levine A, Zagoory-Sharon O, Feldman R, Lewis JG, Weller A (2007). Measuring cortisol in human psychobiological studies. Physiol. Behav..

[9] Holsboer F, Ising M (2009). Stress Hormone Regulation: Biological Role and Translation into Therapy. Annu. Rev. Psychol..

[10] McKenna HT, Reiss IKM, Martin DS (2017). The significance of circadian rhythms and dysrhythmias in critical illness. J. Intensive Care Soc..

[11] McEwen BSC (2002). Cushing’s Syndrome, and a Shrinking Brain—New Evidence for Reversibility. J. Clin. Endocrinol. Metab..

[12] Turpeinen U (2013). & Hämäläinen, Esa (HUSLAB, Laboratory of Women’s Clinic, Haartmaninkatu 2, 00290 Helsinki, F. Determination of cortisol in serum, saliva and urine. Best Pract. Res. Clin. Endocrinol. Metab..

[13] Kinnamon David, Muthukumar Sriram, Panneer Selvam Anjan, Prasad Shalini (2017). Portable Chronic Alcohol Consumption Monitor in Human Sweat through Square-Wave Voltammetry. SLAS TECHNOLOGY: Translating Life Sciences Innovation.

[14] Vabbina PK, Kaushik A, Pokhrel N, Bhansali S, Pala N (2015). Electrochemical cortisol immunosensors based on sonochemically synthesized zinc oxide 1D nanorods and 2D nanoflakes. Biosens. Bioelectron..

[15] Frasconi M, Mazzarino M, Botrè F, Mazzei F (2009). Surface plasmon resonance immunosensor for cortisol and cortisone determination. Anal. Bioanal. Chem..

[16] Kinnamon D, Ghanta R, Lin K-C, Muthukumar S, Prasad S (2017). Portable biosensor for monitoring cortisol in low-volume perspired human sweat. Sci. Rep..

[17] Parlak Onur, Keene Scott Tom, Marais Andrew, Curto Vincenzo F., Salleo Alberto (2018). Molecularly selective nanoporous membrane-based wearable organic electrochemical device for noninvasive cortisol sensing. Science Advances.

[18] Benedetto A, Ballone P (2016). Room temperature ionic liquids interacting with bio-molecules: an overview of experimental and computational studies. Philos. Mag..

[19] Munje RD, Muthukumar S, Jagannath B, Prasad S (2017). A new paradigm in sweat based wearable diagnostics biosensors using Room Temperature Ionic Liquids (RTILs). Sci. Rep..

[20] Jagannath Badrinath, Muthukumar Sriram, Prasad Shalini (2018). Electrical double layer modulation of hybrid room temperature ionic liquid/aqueous buffer interface for enhanced sweat based biosensing. Analytica Chimica Acta.

[21] Diana, C., Hermann, W. & Christian, H. Protein Denaturation by Ionic Liquids and the Hofmeister Series: A Case Study of Aqueous Solutions of Ribonuclease A. *Angew. Chemie Int. Ed*. **46**, 8887–8889 (2007). 10.1002/anie.200702295 17939144

[22] Dickinson EJF, Ekström H, Fontes E (2014). COMSOL Multiphysics®: Finite element software for electrochemical analysis. A mini-review. Electrochem. commun..

[23] Pryor, R. W. *Multiphysics modeling using COMSOL: a first principles approach*. (Jones & Bartlett Publishers, 2009).

[24] Pandiyaraj KN, Selvarajan V, Deshmukh RR, Bousmina M (2008). The effect of glow discharge plasma on the surface properties of Poly (ethylene terephthalate) (PET) film. Surf. Coatings Technol..

[25] Bard, A. J., Faulkner, L. R., Leddy, J. & Zoski, C. G. *Electrochemical methods: fundamentals and applications*. **2** (wiley New York, 1980).

[26] Lim CY (2014). Succinimidyl Ester Surface Chemistry: Implications of the Competition between Aminolysis and Hydrolysis on Covalent Protein Immobilization. Langmuir.

[27] Brust M (2013). Rheology of Human Blood Plasma: Viscoelastic Versus Newtonian Behavior. Phys. Rev. Lett..

[28] Park S-M, Yoo J-S (2003). Peer Reviewed: Electrochemical Impedance Spectroscopy for Better Electrochemical Measurements. Anal. Chem..

[29] K’Owino IO, Sadik OA (2005). Impedance Spectroscopy: A Powerful Tool for Rapid Biomolecular Screening and Cell Culture Monitoring. Electroanalysis.

[30] Perkin S (2012). Ionic liquids in confined geometries. Phys. Chem. Chem. Phys..

[31] Hayes R (2011). Double Layer Structure of Ionic Liquids at the Au(111) Electrode Interface: An Atomic Force Microscopy Investigation. J. Phys. Chem. C.

[32] Islam MM, Alam MT, Ohsaka T (2008). Electrical Double-Layer Structure in Ionic Liquids: A Corroboration of the Theoretical Model by Experimental Results. J. Phys. Chem. C.

[33] Akdogan Y, Junk MJN, Hinderberger D (2011). Effect of Ionic Liquids on the Solution Structure of Human Serum Albumin. Biomacromolecules.

[34] Dorn LD, Lucke JF, Loucks TL, Berga SL (2007). Salivary cortisol reflects serum cortisol: analysis of circadian profiles. Ann. Clin. Biochem..

[35] Mckay L. I., Cidlowski J. A. *Holland-Frei Cancer Medicine*. (Hamilton (ON): BC Decker; 2003, 2003).

[36] Jia W (2013). Electrochemical Tattoo Biosensors for Real-Time Noninvasive Lactate Monitoring in Human Perspiration. Anal. Chem..

[37] Stevens RC, Soelberg SD, Near S, Furlong CE (2008). Detection of cortisol in saliva with a flow-filtered, portable surface plasmon resonance biosensor system. Anal. Chem..

[38] Liu X (2011). Detection of cortisol at a gold nanoparticle|Protein G-DTBP-scaffold modified electrochemical immunosensor. Analyst.

[39] Munje RD, Muthukumar S, Panneer Selvam A, Prasad S (2015). Flexible nanoporous tunable electrical double layer biosensors for sweat diagnostics. Sci. Rep..

[40] Zangheri M (2015). A simple and compact smartphone accessory for quantitative chemiluminescence-based lateral flow immunoassay for salivary cortisol detection. Biosens. Bioelectron..

