# Supplementary material for: Development of ultra-low volume, multi-bio fluid, cortisol sensing platform
Source: Sci Rep. 2018 Nov 13;8:16745. doi: 10.1038/s41598-018-35199-5 (PMC6233171; doi:10.1038/s41598-018-35199-5)
Supplement: Supplementary file 1 — Supplementary Information [file 41598_2018_35199_MOESM1_ESM.docx]

***“*Development of ultra-low volume, multi-bio fluid, cortisol sensing platform*”***

*Sayali Upasham^1^, Ambalika Tanak^1^, Badrinath Jagannath^1^, Dr. Shalini Prasad^1*^*

*^1^Deparatment of Bioengineering, University of Texas at Dallas, T.X-75080, USA.*

*Correspondence: Dr. Shalini Prasad, [Shalini.Prasad@utdallas.edu](mailto:Shalini.Prasad@utdallas.edu)

**Supplementary information :**

**
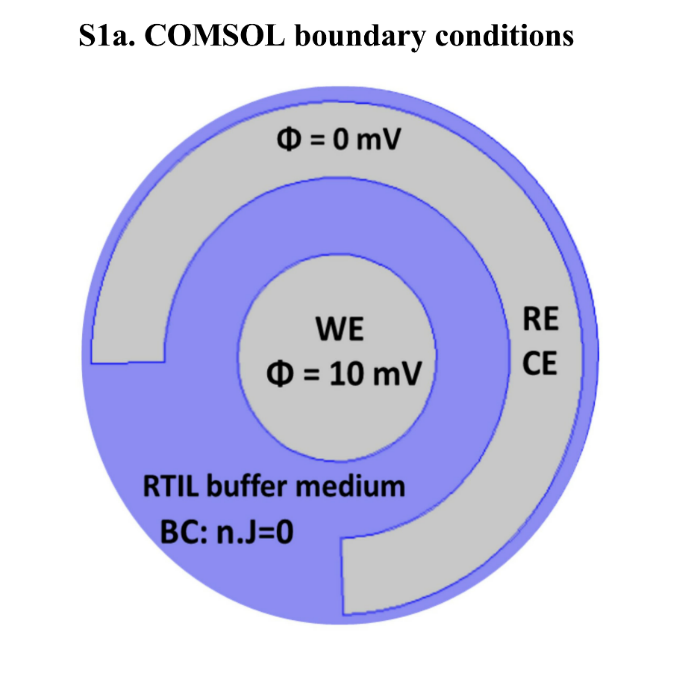
**

**S1b.**


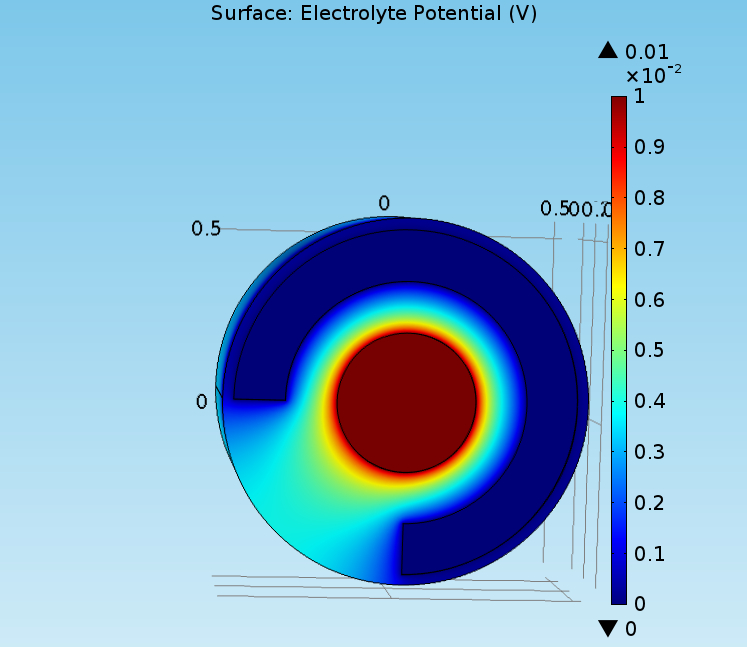


**S1c.**


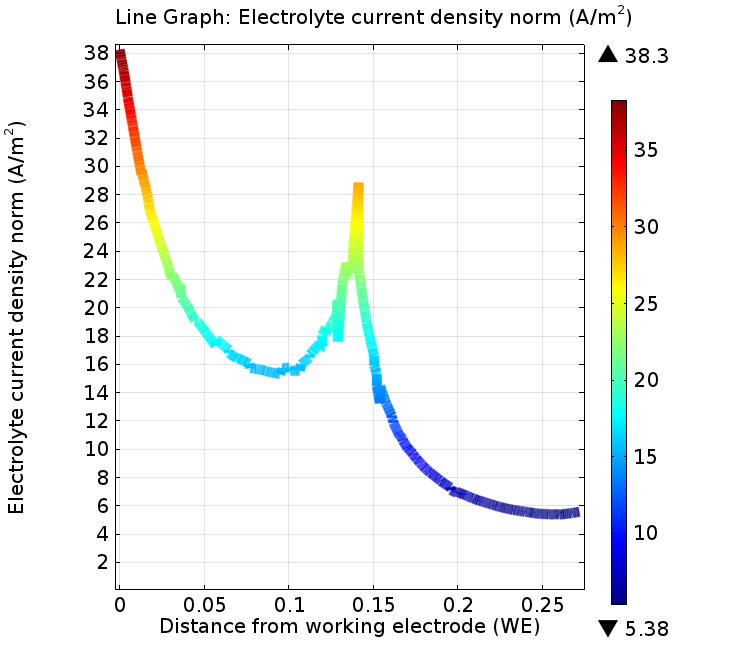


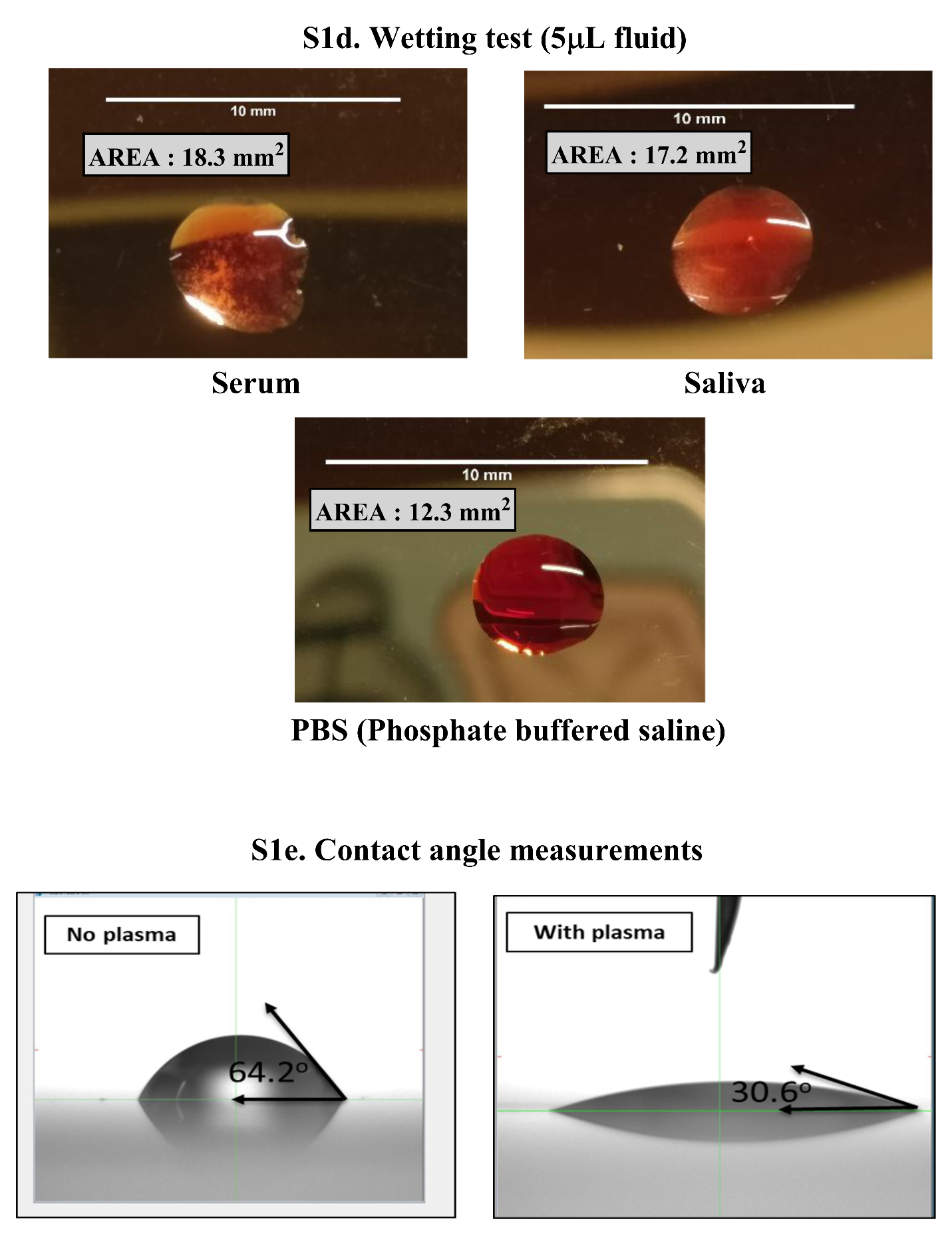


**Figure S1.** **(a)** Depiction of the boundary conditions for the electrode geometry applied during COMSOL Multiphysics® software simulations. **(b)(c)** Simulation plots for **(b)** potential and **(c)** current density for scaled down electrode. **(d)** Wetting test to calculate uniform wicking area for 5μL volume of bio-fluid on PET substrate. **(e)** Contact angle measurements with and without plasma treatment for enhancing hydrophilicity of the surface, which is indicated by drop in contact angle from 64.2 ^o^ to 30.6^o^.

1. **COMSOL Multiphysics software equations**

Equations used in the COMSOL Multiphysics software simulations that govern the simulated potential and current density are follows:

1. $\nabla.$J_l_ = Q_l_ , J_l_ = -σ_l_ $\nabla\phi$_l_

2.$\nabla.$J_l_ = Q_s_ , J_s_ = -σ_s_ $\nabla\phi$_s_

3.$\phi$_s_ - $\phi$_l_ = E_eq_

J_s_ and J_l_ are the current density vectors (A/m^2^) for the electrode and electrolyte respectively. σ_s_ and σ_l_ are conductivities of electrolyte and electrode domains. E_eq_ depicts the equilibrium potential difference at the electrode-electrolyte interface.^1^

1. **FTIR extended analysis.**

Amide bond-I is visible as a peak in the 1500-1550 cm^-1^ region, which is indicative of the secondary structure content of the protein^2^. These peaks which are located at 1558, 1540 and 1506 cm^-1^ are mainly due to the stretching vibrations of the C=O bonds of the amino acids. Amide-II bond visible as a peak at 1652 cm^-1^, arises due to the bending vibration of the N-H bond present in the antibody. It is an indicator of the secondary content of the protein^3^. In addition to this, the CH_2_ bending is visible as a peak at 1458 cm^-1^ is caused due to the symmetrical bending vibrations of the sidechain present in the protein structure or the antibody structure^4^.

1. **Fitting of data using Z-view**

The data for serum (invasively obtained bio-fluid) and sweat (non-invasively obtained bio-fluid) was fitted using a modified Randle’s circuit with the following components:


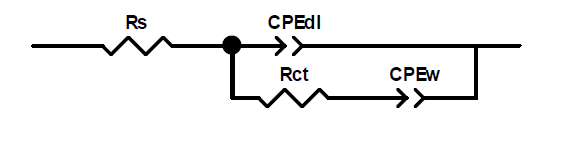


Rs is solution resistance which is contributed from the bulk solution, CPEdl is the Constant phase element which was used to model the capacitive changes in the electrical double layer. Rct is the charge transfer resistance which is the resistance offered to transfer of charges when there are insulative, immobilized biomolecules on the surface and CPEw is the Warburg impedance which is an artifact of diffusion. The following table are the values for the fitting paarmeters, where the highlighted column is the component that is being modulated with cortisol binding to the capture probe.

| **Human serum** | | | |
| --- | --- | --- | --- |
| **Doses** | **Rs** | **Rct** | **Cedl** |
| Baseline | 222.4 | 5.75E+06 | 4.63E-08 |
| 10 ng/ml | 428.8 | 6.09E+06 | 3.0983E-08 |
| 50 ng/ml | 444.3 | 7.56E+06 | 2.97E-08 |
| 100 ng/ml | 433.8 | 8.15E+06 | 2.96E-08 |
| 150 ng/ml | 450.9 | 8.35E+06 | 2.95E-08 |
| 200 ng/ml | 479 | 8.48E+06 | 2.89E-08 |
| 300 ng/ml | 500.2 | 8.79E+06 | 2.75E-08 |
| **Human sweat** | | | |
| **Doses** | **Rs** | **Rct** | **Cedl** |
| Baseline | 174 | 2.75E+06 | 7.04E-08 |
| 5 ng/ml | 416.3 | 3.00E+06 | 6.19E-08 |
| 10 ng/ml | 563.2 | 3.40E+06 | 5.52E-08 |
| 50 ng/ml | 592.1 | 3.65E+06 | 5.09E-08 |
| 100 ng/ml | 602.6 | 4.15E+06 | 4.90E-08 |
| 150 ng/ml | 564.6 | 4.50E+06 | 4.73E-08 |
| 200 ng/ml | 637 | 4.89E+06 | 4.69E-08 |

**4. Regression analysis for sensor response**


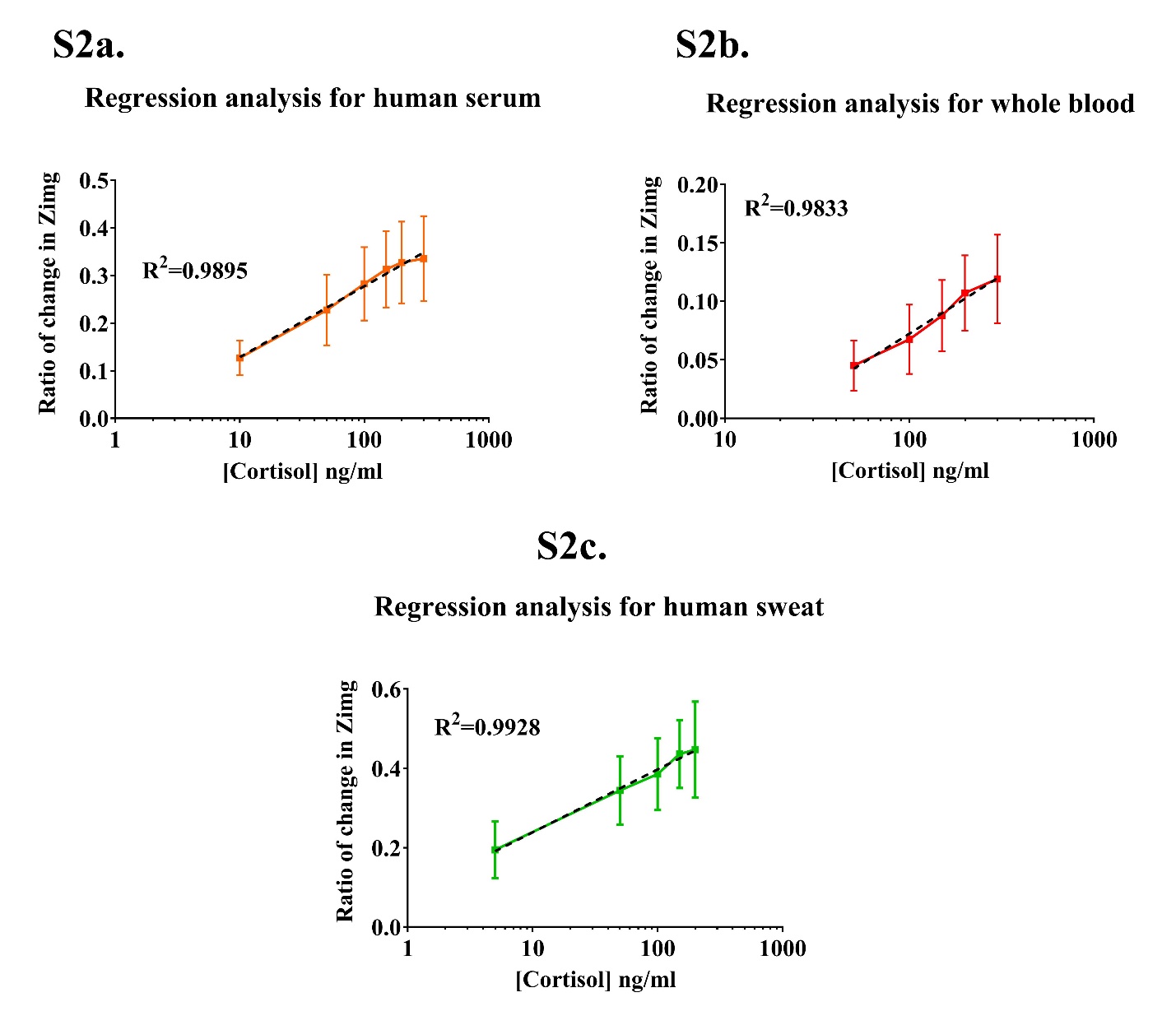


**Figure S2. (a)** Regression analysis for dose response in serum. **(b)** Regression analysis for dose response in whole blood. **(c)** Regression analysis for dose response in sweat.


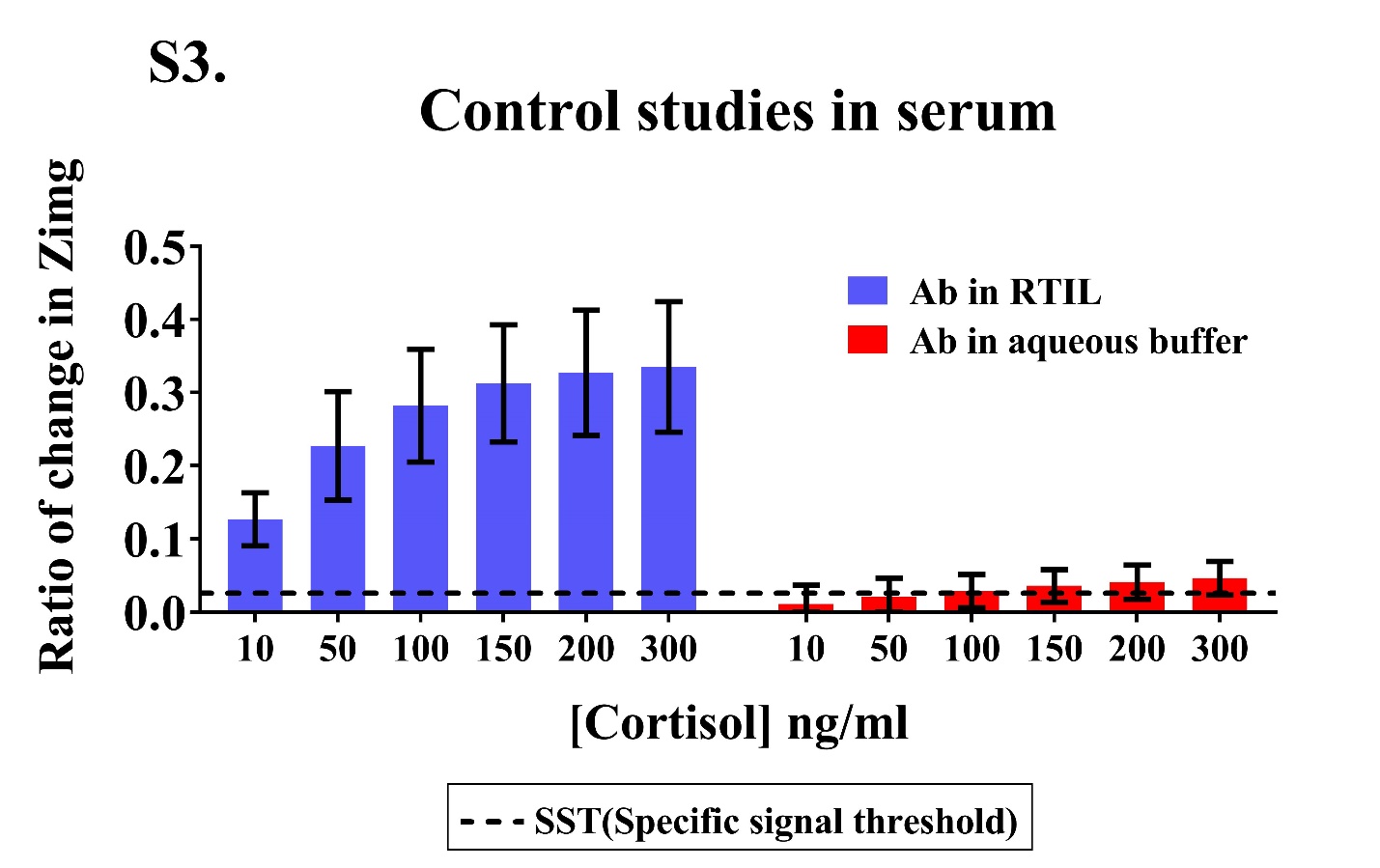


**Figure S3. Control studies in serum**

Performance of the sensor in the presence of RTIL was compared against performance in aqueous buffer. Signal response for RTIL is higher than that of the latter indicating its suitability to be used for electrochemical detection of cortisol for enhancing signal response.

.

**Figure S4. (a)** Control study for cortisol in saliva **(b)** Saturation curve for antibody incubation time -Optimization control

Blank buffer washes were performed using human saliva to determine the noise threshold. A value of 14% was chosen as the signal-noise threshold for calculating dose response data. Phase response curve saturates at 90 minutes which was used as the incubation time for antibody.

**
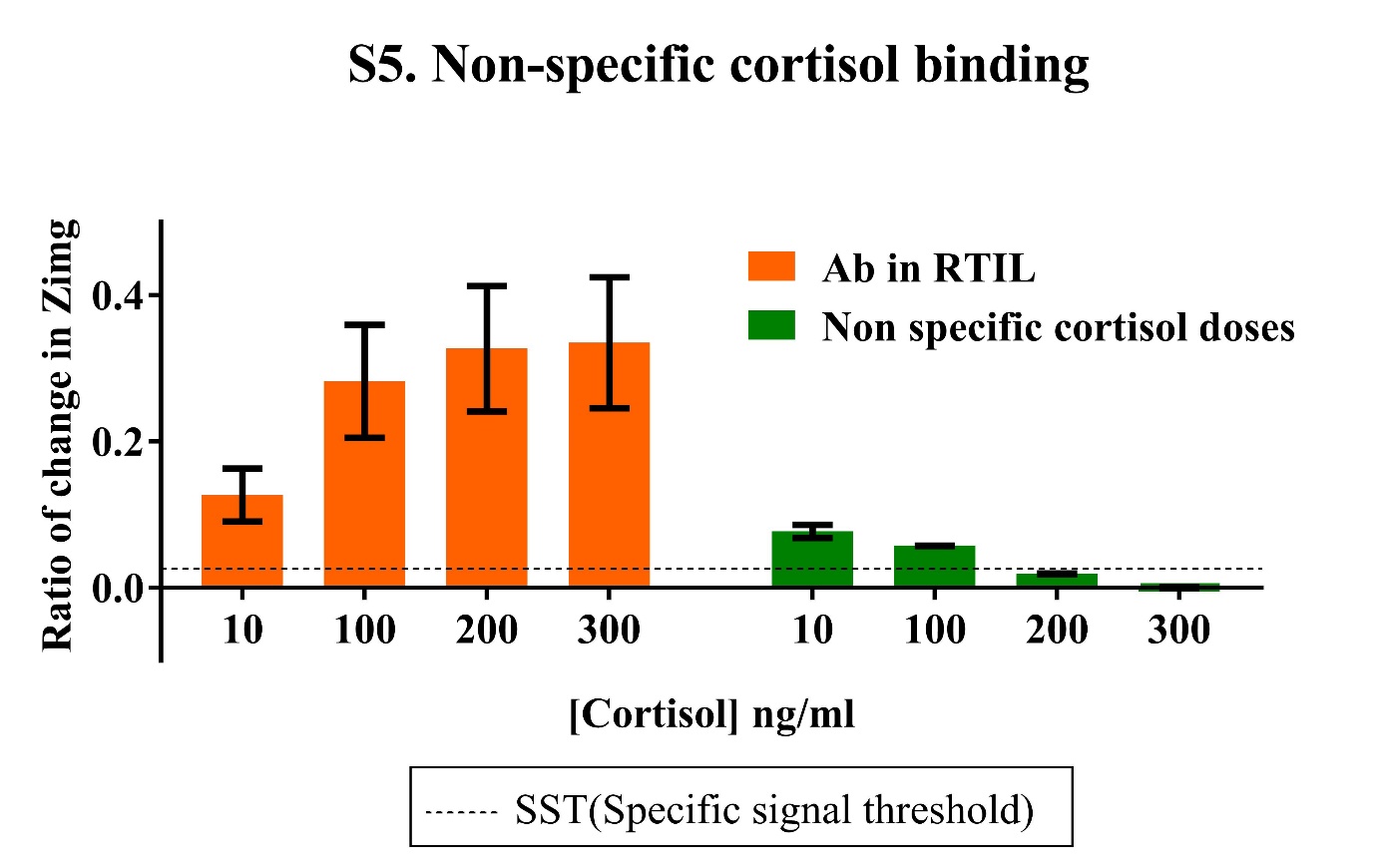
**

**Figure S5: Control experiment to test for non-specific cortisol binding**

Non-specific cortisol binding to the surface was tested by performing a control experiment where unfunctionalized electrode surface was tested with varying concentrations of cortisol. This is depicted in above Fig. S5.

**Figure S6.** Electrode design and dimensions

1. **References**

1. Stevenson, H., Shanmugam, N. R., Selvam, A. P. & Prasad, S. The Anatomy of a Nonfaradaic Electrochemical Biosensor. *SLAS Technol. Transl. Life Sci. Innov.* **23,** 5–15 (2017).

2. Diana, C., Hermann, W. & Christian, H. Protein Denaturation by Ionic Liquids and the Hofmeister Series: A Case Study of Aqueous Solutions of Ribonuclease A. *Angew. Chemie Int. Ed.* **46,** 8887–8889 (2007).

3. Lim, C. Y. *et al.* Succinimidyl Ester Surface Chemistry: Implications of the Competition between Aminolysis and Hydrolysis on Covalent Protein Immobilization. *Langmuir* **30,** 12868–12878 (2014).

4. Susi, H. & Michael Byler, D. Protein structure by Fourier transform infrared spectroscopy: Second derivative spectra. *Biochem. Biophys. Res. Commun.* **115,** 391–397 (1983).
